# Development of a Genetically Modified *Lactococcus lactis* Strain that Produces a Single-Chain Variable Fragment Targeting Interleukin-6 Receptor α to Suppress Serum Amyloid A

**DOI:** 10.1007/s00284-025-04518-1

**Published:** 2025-09-26

**Authors:** Masahiro Yoda, Kaho Suzuki, Shoko Yoda, Kazuma Inoue, Mao Kagotani, Misato Kubo, Aito Murakami, Fu Namai, Takashi Sato, Takeshi Shimosato

**Affiliations:** 1 Graduate School of Medicine, Science and Technology, Shinshu University, Nagano, 399-4598 Japan; 2 Graduate School of Science and Technology, Shinshu University, 8304 Minamiminowa, Kamiina, Nagano, 399-4598 Japan; 3 Institute for Aqua Regeneration, Shinshu University, Nagano, 399-4598 Japan; 4 Food and Feed Immunology Group, Laboratory of Animal Food Function, Graduate School of Agricultural Science, Tohoku University, Sendai, Miyagi 980-8572 Japan; 5 Institute for Biomedical Sciences, Shinshu University, Nagano, 399-4598 Japan

## Abstract

Interleukin-6 (IL-6) is a pivotal cytokine in immune regulation, inflammatory responses, and chronic inflammation. Dysregulated IL-6 signaling, which results from the interaction between IL-6 and IL-6 receptor alpha (IL-6Rα), has been implicated in the pathogenesis of numerous inflammatory diseases. Although anti-IL-6Rα monoclonal antibodies have been used to treat these diseases, their high treatment costs impose a substantial financial burden on patients. Recently, a genetically engineered strain of *Lactococcus lactis* (gmLAB) has attracted attention as a low-cost therapeutic agent. Thus, in this study, as a lower-cost alternative, a novel strategy using a gmLAB to produce a single-chain variable fragment targeting IL-6Rα (IL-6RαscFv) was developed. A recombinant strain that produces IL-6RαscFv with high binding affinity for human IL-6Rα, effectively inhibiting the interaction between IL-6 and IL-6Rα, was successfully produced. Furthermore, IL-6RαscFv significantly suppressed the expression of serum amyloid A, a biomarker of acute-phase inflammation. These findings demonstrate the potential of the recombinant strain as a promising therapeutic approach for inflammatory diseases.

**Supplementary Information:**

The online version contains supplementary material available at 10.1007/s00284-025-04518-1.

## Introduction

Interleukin-6 (IL-6) is a pleiotropic proinflammatory cytokine that is essential in host immunity because it stimulates various biochemical functions [1]. IL-6 initially binds to its cognate receptor, IL-6 receptor alpha (IL-6Rα), to form a complex that subsequently associates with the signal-transducing molecule gp130 [2]. This association with gp130 activates downstream signaling mechanisms that facilitate diverse biological processes, including an increase of acute-phase proteins, such as serum amyloid A (SAA). Though IL-6 is essential for host defense, its overexpression has been implicated in the pathology of various diseases, including cancer [3–6], multiple sclerosis [7], rheumatoid arthritis [8], diabetes mellitus, anemia [9], inflammatory bowel disease [10], and Alzheimer’s disease [11]. Thus, IL-6 signaling is regarded as a key therapeutic target.

Anti-IL-6Rα monoclonal antibodies (mAbs) have been widely used to manage IL-6-mediated conditions. For instance, sarilumab, a fully humanized anti-IL-6Rα mAb [12], binds to IL-6Rα with high affinity and thereby blocks the IL-6-mediated inflammatory signaling cascade. Similarly, other mAbs, such as tocilizumab [13], sirukumab [14], olokizumab [15], and siltuximab [16], have demonstrated therapeutic benefits. Despite their clinical success, these mAbs have several disadvantages, including their high costs. Thus, there is a need to develop alternative IL-6 inhibitors that are more cost-effective.

Single-chain variable fragments (scFvs), which are small recombinant antibodies (Abs) capable of antigen binding, are a promising alternative to anti-IL-6Rα mAbs. They consist of the variable regions, which are the smallest functional units of an Ab that can bind an antigen, of the heavy (VH) and light (VL) chains [17, 18]. These scFvs offer several advantages, including faster clearance from the bloodstream and reduced immunogenicity than intact immunoglobulin (Ig) G molecules [19]. Due to advances in bacterial expression systems, scFvs can be efficiently produced in bacteria, and production is scalable. Recent developments in cell engineering have further expanded the potential of genetically modified lactic acid bacteria (gmLABs) as platforms for biomedical protein production [20, 21]; gmLABs have been successfully used to produce various therapeutic proteins [22–24], including scFvs [25]. In addition, the mucosal delivery of proteins using gmLABs has been reported to be an efficient and low-cost therapeutic approach [26]. These features make scFv-producing gmLABs an attractive option for the treatment of IL-6-related diseases. Previously, a gmLAB strain was engineered to produce a mouse IL-6-targeting scFv (mIL-6scFv), which demonstrated immunoreactivity against mIL-6 [23]. In the present study, a novel gmLAB strain capable of producing an scFv targeting IL-6Rα (IL-6RαscFv) was developed. Although both approaches aim to inhibit the IL-6/IL-6Rα signaling complex and thereby suppress inflammation, the target molecules differ entirely, IL-6 in the former case, and IL-6Rα in the present study.

The aim of this study was to develop a cost-effective alternative to anti-IL-6Rα monoclonal antibodies by engineering a gmLAB to produce an IL-6RαscFv. A lactococcal expression system to produce rIL-6RαscFv under the control of a nisin-inducible promoter was constructed. Its expression, antigen-binding activity against human and mouse IL-6Rα, and functional efficacy in inhibiting IL-6 signaling were evaluated. The present findings highlight the potential of IL-6RαscFv-producing *L. lactis* as a novel and affordable therapeutic strategy for IL-6-mediated inflammatory diseases.

## Materials and Methods

### Bacteria, Growth Conditions, and Plasmid

**Fig. 1:**
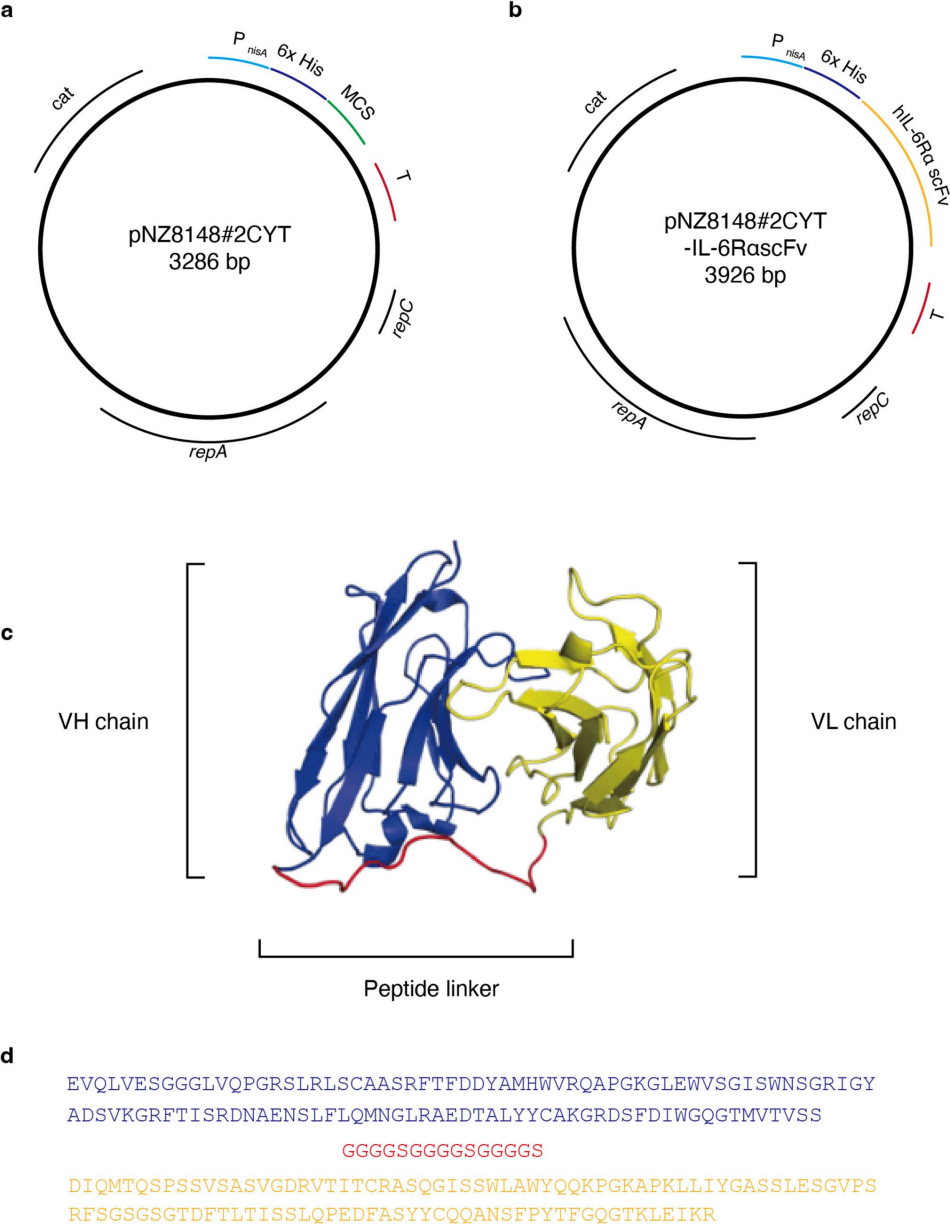
**a**, **b** Schematic representations of the lactococcal gene expression vectors. **a** The gene expression vector pNZ8148#2:CYT. **b** The DNA fragment encoding the *IL-6RαscFv* gene in the MCS of pNZ8148#2:CYT for constructing the IL-6RαscFv expression vector pNZ8148#2:CYT-IL-6RαscFv. *P*_*nisA*_: nisin-inducible promoter, *6* × *His*: 6 × histidine tag, *MCS*: multi-cloning site, *T*: terminator, *repC and repA*: the origins of replication, *cat*: chloramphenicol acetyltransferase gene. **c**, **d** Predicted model and amino acid sequence of the designed rIL-6RαscFv. **c** Predicted 3D model of rIL-6RαscFv built using SWISS-MODEL. The locations of the VH chain (blue), the VL chain (yellow), and the peptide linker (red) are shown. VH chain: variable region of the heavy chain, VL chain: variable region of the light chain. **d** The designed amino acid sequence of rIL-6RαscFv. The sequences of the VH chain, the VL chain, and the peptide linker are shown in blue, yellow, and red, respectively

The host strain NZ9000 (MobiTec, Goettingen, Germany), which is a derivative of *L. lactis* subsp. *cremoris* MG1363, was used. NZ9000 is a standard host strain for nisin-regulated gene expression (NICE) [27]. M17 broth (BD Difco; Becton, Dickinson and Company, Sparks, MD, USA) with 0.5% (w/v) glucose (GM17) was used as the bacterial culture medium. The constructed genetically modified strains were cultured in GM17 containing 10 μg/mL chloramphenicol (GM17cm) at 30 ℃. The IL-6RαscFv gene was expressed using the intracellular-accumulating expression vector, pNZ8148#2CYT [28] (Fig. 1a, b), which is suitable for the NICE system.

### Protein Structure and Construction of gmNZ9000 Strains for IL-6RαscFv Gene Expression

The amino acid sequence of the complementarity-determining regions of both the VH and VL chains of sarilumab, which has been marketed as a recombinant human IgG1 mAb against IL-6Rα [29], was used, and the VL and VH chains were connected by a peptide linker, GGGGSGGGGSGGGGS. The three-dimensional (3D) structure of the designed scFv was predicted based on the common scFv structure using SWISS-MODEL (Fig. 1c). Figures were prepared using Pymol [30]. The designed amino acid sequence of rIL-6RαscFv is shown in Fig. 1d. General molecular cloning techniques were performed according to previously described methods [31]. The resulting plasmid sequence data were deposited in DNA Data Bank of Japan (DDBJ) (pNZ8148#2:CYT-IL-6RαscFv; DDBJ accession number: LC853333) and were confirmed to be consistent with the predicted sequence. Then, pNZ8148#2:CYT-IL-6RαscFv was introduced into NZ9000 cells by electroporation using a Gene Pulser Xcell electroporation system (Bio-Rad, Hercules, CA, USA). The resulting gmNZ9000 strain was designated NZ-IL6RαscFv. NZ-VC [32], which is a pNZ8148#2:CYT-harboring strain, was used as a vector control strain.

### Induction of Recombinant Gene Expression in the gmNZ9000 Strains and Sample Preparation

After overnight incubation, the gmNZ9000 strains were inoculated at a dilution of 1/20 into fresh GM17cm that had been pre-heated to 30 ℃. The gmNZ9000 strains were then cultured in 2 mL, 50 mL, and 4 L for sodium dodecyl sulfate–polyacrylamide gel electrophoresis (SDS-PAGE) and western blotting, assay for immunoreactivity, and purification of the scFv, respectively. The cultures were then incubated until the turbidity reached an optical density at 600 nm (OD_600_) of approximately 0.4. Subsequently, the cultures were supplemented with nisin (MoBiTec) at 1.25 ng/mL and incubated for 3 h. After nisin induction, bacterial cells were separated by centrifugation at 8000 × *g* and 4 ℃ for 5 min. Bacterial cells from 2 to 50-mL cultures were washed once with ice-cold Tris-buffered saline (TBS; 50 mM Tris, 138 mM sodium chloride, 2.7 mM potassium chloride, pH 8.0), then suspended in 400 μL of ice-cold TBS-containing protease inhibitor cocktail (Roche Diagnostics, Mannheim, Germany). The suspension was mixed with 0.4 g of glass beads (0.2 mm in diameter, AS ONE, Osaka, Japan), and shaken using a bead beater (lT-12, TAITEC, Saitama, Japan). The soluble fraction was collected by centrifugation at 20,400 × *g* and 4 ℃ for 15 min. An equal volume of 2 × sample buffer solution (196–11022, Wako, Osaka, Japan) was added to some of the samples, and the mixtures were boiled for 5 min for SDS-PAGE. Cells from 4-L cultures were collected by centrifugation at 8000 × g and 4 ℃ for 5 min and washed twice with ice-cold phosphate-buffered saline (PBS; 137 mM sodium chloride, 2.7 mM potassium chloride, 10 mM disodium hydrogen phosphate, 1.76 mM potassium dihydrogen phosphate, pH 7.4). The frozen-cellular pellet was ground with aluminum oxide powder (Wako Pure Chemical Industries, Ltd.) (pellet:Al2O3 = 1:2) on a mortar for 15 min on ice. The homogenate was collected with ice-cold lysis buffer (20 mM sodium phosphate, 500 mM sodium chloride, and 20 mM imidazole; pH 7.4) containing an ethylenediaminetetraacetic acid (EDTA) free-protease inhibitor cocktail tablet (Roche Diagnostics GmbH, Mannheim, Germany), and it was clarified by two-time centrifugation at 20,400 × g and 4 ℃ for 20 min.

### Detection of rIL-6RαscFv

The protein samples were analyzed by SDS-PAGE with a 15% (v/v) polyacrylamide gel and immunoblotting with mouse anti-His-tag Ab (BioLegend, San Diego, CA, USA) and horseradish peroxidase (HRP)-conjugated goat anti-mouse IgG (Sigma-Aldrich, St. Louis, MO, USA). The blots were reacted with ECL Prime Western Blotting Detection Reagent (Cytiva, Marlborough, MA, USA) and detected using a Lumino image analyzer (ImageQuant LAS 500; Cytiva). In addition, to determine the level of rIL-6RαscFv at each time point (1, 3, 6, 12, 24, and 48 h), samples were prepared as described above, and blots were conducted.

### Assay for the Immunoreactivity of NZ-IL6RαscFv to hIL-6Rα and mIL-6Rα

NZ-IL6RαscFv and NZ-VC cellular extracts were prepared from samples incubated for 3 h after nisin induction in 50 mL of GM17cm. The total protein concentration of each extract was measured using a BCA Protein Assay kit (Thermo Fisher Scientific, Rockford, IL, USA) according to the manufacturer’s instructions and adjusted to 2 mg/mL, and the extracts were then serially diluted in twofold steps using PBS. The immunoreactivity of rIL-6RαscFv was examined using a enzyme-linked immunosorbent assay (ELISA) [22, 23]. 50 μL of 500 ng/mL of soluble recombinant human IL-6Rα (hIL-6Rα) (BioLegend, Cat #: 751504) or of soluble recombinant mouse IL-6Rα (mIL-6Rα) (BioLegend, Cat #: 795204) were immobilized on a 96-well plate (Thermo Fisher Scientific) and incubated at 4 ℃ overnight. In addition, non-immobilized wells were also used to confirm the extent to which rIL-6RαscFv stuck to the bottom of the plate. Each well was washed three times with 200 μL of phosphate-buffered saline (PBS) containing 0.05% Tween 20 (Nacalai Tesque) (PBS-T), and then 100 μL of 1% bovine serum albumin in PBS were added to each well. After 2 h of incubation at room temperature (RT), each well was washed with PBS-T, and 50 μL of the prepared protein samples were added. After 2 h of incubation at RT, each well was washed, and 50 μL of 1/1000 anti-His tag antibody (Biolegend) were added. After 2 h incubation at RT, each well was washed, and 50 μL of a 1/3000 dilution of peroxidase-conjugated anti-mouse IgG (Biolegend) were added. After 1 h of incubation at RT, each well was washed, and 50 μL of 3,3,5,5-tetra-methylbenzidine were added. After 10 min of incubation at RT, the reaction was stopped by the addition of 50 μL 2 N sulfuric acid, and the absorbance at 450 nm was measured using an iMark microplate reader (Bio-Rad).

### Sequence Alignment of hIL-6Rα and mIL-6Rα

To clarify the differences in the binding properties of human and mouse IL-6Rα to sarilumab, the amino acid sequences of the human IL-6Rα (UniProt accession: P08887) and the mouse IL-6Rα (UniProt accession: P22272) were aligned using clustal omega (https://www.ebi.ac.uk/jdispatcher/msa/clustalo?stype=protein).

### Purification of IL-6RαscFv

The rIL-6RαscFv was purified using a HisTrap HP column (1 mL; Cytiva) and a fast protein liquid chromatography system (AKTA pure 25; Cytiva). The filtrate was loaded onto the column equilibrated with 1 × binding buffer (20 mM sodium phosphate and 500 mM sodium chloride; pH 7.4), the column was then washed sequentially with binding buffer, and rIL-6RαscFv was eluted with a linear gradient of 0 to 500 mM imidazole and dialyzed against PBS. The concentration of rIL-6RαscFv in the dialyzed sample was measured using a His Tag ELISA Detection Kit (Funakoshi, Tokyo, Japan). The dialyzed samples were subsequently used in the following inhibition and bioactivity assays.

### IL-6-binding Inhibition Assay

The ability of rIL-6RαscFv to prevent IL-6 from binding to IL-6Rα was examined using an ELISA. First, 100 μL of 500 ng/mL recombinant hIL-6Rα (BioLegend) were immobilized on a 96-well plate and incubated overnight at 4 ℃. After the wells were washed, 100 μL of 1% (w/v) bovine serum albumin in PBS were added to each well, and the samples were incubated at RT for 2 h. The wells were then washed, 50 μL of the 25 ng/mL rIL-6RαscFv that were serially diluted in fivefold steps (0.2, 1, 5, 25 ng/mL) were added, and the samples were incubated for 30 min. Sarilumab (Selleck Chemicals, Houston, TX, USA) (0.001, 0.01, 0.1, 1, 10, 100 μg/mL), and purified human IgG (normal human IgG, whole molecule, purified: Fujifilm Wako Pure Chemicals, Osaka, Japan) (0.001, 0.01, 0.1, 1, 10, 100 μg/mL) diluted in PBS were used as a positive control and negative control, respectively, to demonstrate that the inhibition assay was established. Next, 50 μL of 1000 ng/mL hIL-6 (BioLegend) were added to each well. Non-immobilized wells were also used to confirm no irrelevant reactions. The plate was shaken for 10 min and then incubated at RT for 2 h, after which the wells were washed, 100 μL of the detection Ab solution (Human IL-6 ELISA Max Detection Ab; BioLegend) were added, and the samples were incubated at RT for 1 h. The wells were then washed, 100 μL of avidin-HRP were added, and the samples were incubated for 30 min at RT. Subsequently, the wells were washed, and 100 μL of the HRP substrate were added. After 10 min of incubation at RT, the reaction was stopped by the addition of 100 μL of 2 N sulfuric acid.

### Culture Conditions for HepG2 Cells

HepG2 human liver carcinoma cells (Cellular Engineering Technologies, Coralville, IA, USA) were cultured in 10-cm cell culture dishes (Techno Plastic Products AG, Trasadingen, Switzerland) with Human Hepatocellular Carcinoma Expansion Media (Cellular Engineering Technologies) containing 10% (v/v) fetal bovine serum (GE Healthcare), 100 U/mL penicillin, and 100 μg/mL streptomycin (Nacalai Tesque, Kyoto, Japan) at 37 ℃ in a humidified incubator with 5% carbon dioxide.

### Inflammation Inhibition Assay of the Purified IL-6RαscFv in HepG2 Cells

HepG2 cells were seeded onto 24-well plates (Nippon Genetics, Tokyo, Japan) at 1 × 10^5^ cells/well and cultured for 4 days. To determine the optimized hIL-6 (Biolegend) concentration for *SAA1* expression, the cells were incubated with 0, 12.5, 25, 50, or 100 ng/mL of IL-6. In the inflammation inhibition assay, the cells were incubated for 30 min with purified rIL-6RαscFv (1 or 10 ng/mL), sarilumab (Selleck Chemicals)(1000 ng/mL) as a positive control, and purified human IgG (Fujifilm Wako Pure Chemicals) 1000ng/mL) as a negative control in culture media; 0.34 nM of rIL-6RαscFv (MW: 29.1 kDa) and sarilumab (MW: 144.13 kDa) were also used for the inhibition assay to compare the effectiveness of the two molecules. After 30 min of incubation, the HepG2 cells were stimulated with the optimized concentration of hIL-6 for 24 h in culture media.

### RNA Extraction and Real-time Quantitative Reverse Transcription-PCR (RT-qPCR)

Total RNA extraction from the cells and cDNA synthesis were performed using Nucleospin RNA (MACHEREY–NAGEL, Düren, Germany) and a PrimeScript RT reagent kit (Perfect Real Time; Takara Bio, Shiga, Japan). RT-qPCR was performed with a Thermal Cycler Dice Real Time System (Takara Bio) and TB Green Premix Ex Taq II (Takara Bio) according to the manufacturer’s instructions using primers specific for human *SAA1* (Table 1) and β-actin (Takara Bio). The thermal cycling parameters included 45 amplification cycles of 5 s at 95 ℃ for denaturation and 30 s at 60 ℃ for annealing and elongation. Measurements were normalized using the β-actin (housekeeping gene) expression levels.

**Table 1:** Primer sequences

Name	5’-sequence-3’	bp length
*SAA1* Forward	CTGCAGAAGTGATCAGCG	18
*SAA1* Reverse	ATTGTGTACCCTCTCCCC	18

### In Silico* Modeling of the IL-6R/rIL-6RαscFv Complex*

Models of the IL-6Rα/rIL-6RαscFv complex were assembled using the protein–protein docking algorithm Z-DOCK 3.0.2, which examines the statistical potential, shape complementarity, and electrostatics [33–35]. The extracellular domain of the hIL-6Rα structure (Protein Data Bank identifier: 1N26) was used as an initial reference for IL-6Rα model-building, and the rIL-6RαscFv structure was predicted as described above. The top three candidate protein–protein structures obtained using the Z-DOCK scoring function were analyzed [33].

### Statistical Analysis

Statistical analyses were conducted using GraphPad Prism software (Version 7; GraphPad, San Diego, CA, USA). To evaluate the significance of differences, one-way analysis of variance was followed by Tukey–Kramer post hoc tests. Significance was defined as *p* < 0.05. Data are presented as mean ± standard deviation values.

### Availability of Data

The constructed plasmid DNA sequence data that support the findings of this study have been deposited in DDBJ with accession number LC853333.

## Results

### Design of the Anti-IL-6Rα scFv

The 3D structure of the intended scFv (Fig. 1c) was predicted. The nucleotide sequence optimized for *L. lactis* subsp. *cremoris* MG1363 codon usage was converted to the amino acid sequence of the designed scFv (Fig. 1d).

### Construction of the IL-6RαscFv-producing gmLAB, and Confirmation and Optimization of IL-6RαscFv Expression by the gmLAB

**Fig. 2:**
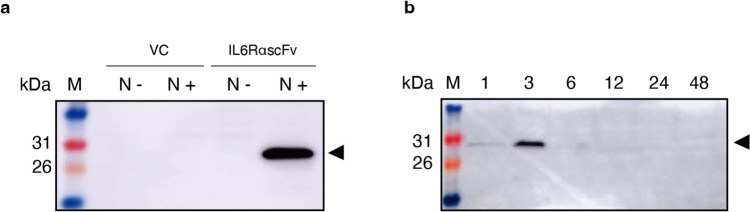
Detection of rIL-6RαscFv and determination of the optimal culture time for rIL-6RαscFv production by NZ-IL6RαscFv. **a** Western blotting of rIL-6RαscFv in the cellular extracts of NZ-VC (VC) and NZ-IL6RαscFv with (N +) or without (N-) nisin. **b** Western blotting of rIL-6RαscFv in the cellular extracts of NZ-VC (VC) and NZ-IL6RαscFv obtained at six time points (1, 3, 6, 12, 24, and 48 h) after the addition of nisin; the time points are indicated at the top. An anti-His-tag Ab was used to detect rIL-6RαscFv. The arrowheads indicate rIL-6αscFv (29.1 kDa). M: molecular mass marker (kDa)

Sequencing of pNZ8148#2:CYT-IL-6RαscFv (Fig. 1b) confirmed that it did not contain any mutations or deletions. In the western blotting experiment using the anti-His-tag Ab, bands corresponding to IL-6RαscFv (29.1 kDa) were detected in the cellular extracts of NZ-IL6RαscFv (Fig. 2a). In contrast, no band was detected in the samples of NZ-VC, and none was detected in the samples without nisin stimulation (Fig. 2a). These results indicated that NZ-IL6RαscFv produced rIL-6RαscFv after nisin stimulation. Next, the optimal culture time for rIL-6RαscFv production was determined. NZ-IL6RαscFv was incubated for 1, 3, 6, 12, 24, or 48 h after nisin induction. After determination of the OD_600_, a cell extract was prepared for each time point, and rIL-6RαscFv expression was detected by western blotting using the anti-His-tag Ab (Fig. 2b). The intensity of the band corresponding to rIL-6RαscFv peaked at 3 h, and it was no longer detectable after 6 h.

### Immunoreactivity of IL-6RαscFv

**Fig. 3:**
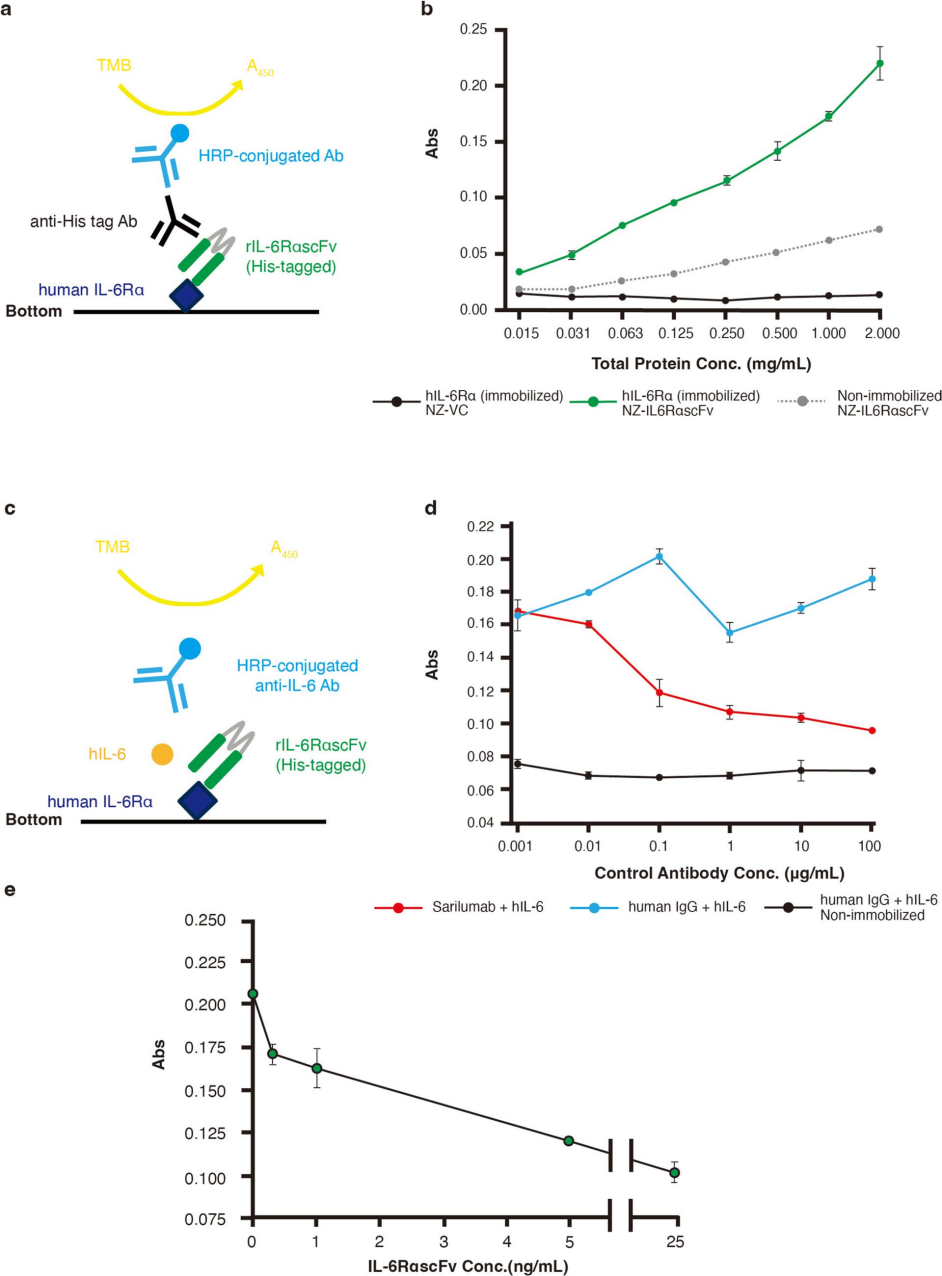
**a**, **b** Immunoreactivity assay of NZ-IL6RαscFv. **a** A schematic representation of the ELISA-based technique. Solid phase-immobilized hIL-6Rα was reacted with serial dilutions of a gmLAB cell extract (NZ-IL6RαscFv or NZ-VC). The hIL-6Rα-binding proteins were then detected using an anti-His-tag Ab. **b** Different concentrations of the cell extract of NZ-IL6RαscFv (green) or NZ-VC (black) were applied to the ELISA system to determine their reactivity to IL-6Rα. In addition, as a control for this assay, the NZ-IL6RαscFv cell extract was added to the wells where hIL-6Rα was not immobilized (dotted gray). **c**, **d**,** e** IL-6-binding inhibition assay of NZ-IL6RαscFv. **c** A schematic representation of the ELISA-based technique. Recombinant hIL-6Rα immobilized on a solid phase was competitively reacted with purified rIL-6RαscFv and recombinant hIL-6. **d** Sarilumab (0.001, 0.01, 0.1, 1, 10, 100 μg/mL) (red) and purified human IgG (0.001, 0.01, 0.1, 1, 10, 100 μg/mL) (blue) were added to hIL-6Rα-immobilized wells as positive and negative controls, respectively, to validate the inhibition assay. Purified human IgG was also applied to hIL-6Rα non-immobilized wells (black). After the samples were incubated for 30 min, 1000 ng/mL hIL-6 were added to each well. The horizontal axis is logarithmic scale with 10 as the base. **e** Various concentrations (0, 0.2, 1, 5, 25 ng/mL) of purified rIL-6RαscFv were applied to the ELISA system to evaluate its inhibitory activity. Each experiment was conducted more than three times, and representative data are shown

To examine whether rIL-6RαscFv was biologically active, the immunoreactivity of rIL-6RαscFv against hIL-6Rα and mIL-6Rα was examined by ELISA. A schematic of the detection method is depicted in Fig. 3a. The results showed that, with hIL-6Rα, the absorbance increased as the total protein concentration of the cellular extracts of NZ-IL6RαscFv increased (Fig. 3b); in contrast, the absorbance remained at the baseline level for all protein concentrations of the cellular extracts of NZ-VC (Fig. 3b). In addition, in the wells without hIL-6Rα immobilization, the absorbance increased slightly as the total protein concentration of the cellular extracts of NZ-IL6RαscFv increased, but the extent was minimal (Fig. 3b). With mIL-6Rα, the absorbance remained at almost the same level as the PBS group for 2.0 mg/mL protein concentrations of the cellular extracts of NZ-IL6RαscFv and NZ-VC (Supplementary material 1).

### Ability of rIL-6RαscFv to Inhibit IL-6 Binding to IL-6Rα

To examine whether IL-6RαscFv inhibits IL-6 binding to IL-6Rα, an inhibition ELISA was performed using purified rIL-6RαscFv. The rIL-6RαscFv was purified (Supplementary material 2), and the concentration of purified rIL-6RαscFv was decided (Supplementary material 3). A schematic of the IL-6-binding inhibition assay is depicted in Fig. 3c. The validity of this inhibition assay was demonstrated using sarilumab and purified human IgG as a positive control and negative control, respectively (Fig. 3d). Compared with human IgG + hIL-6, the absorbance of sarilumab + hIL-6 decreased gradually in a concentration-dependent manner. In addition, the absorbance of purified human IgG + hIL-6 without immobilization of hIL-6Rα remained at the baseline level. The results of the IL-6-binding inhibition assay using rIL-6RαscFv (Fig. 3e) showed that the absorbance decreased gradually in an scFv concentration-dependent manner.

### Confirmation of rIL-6RαscFv Bioactivity in HepG2 Cells and Comparison with Sarilumab

**Fig. 4:**
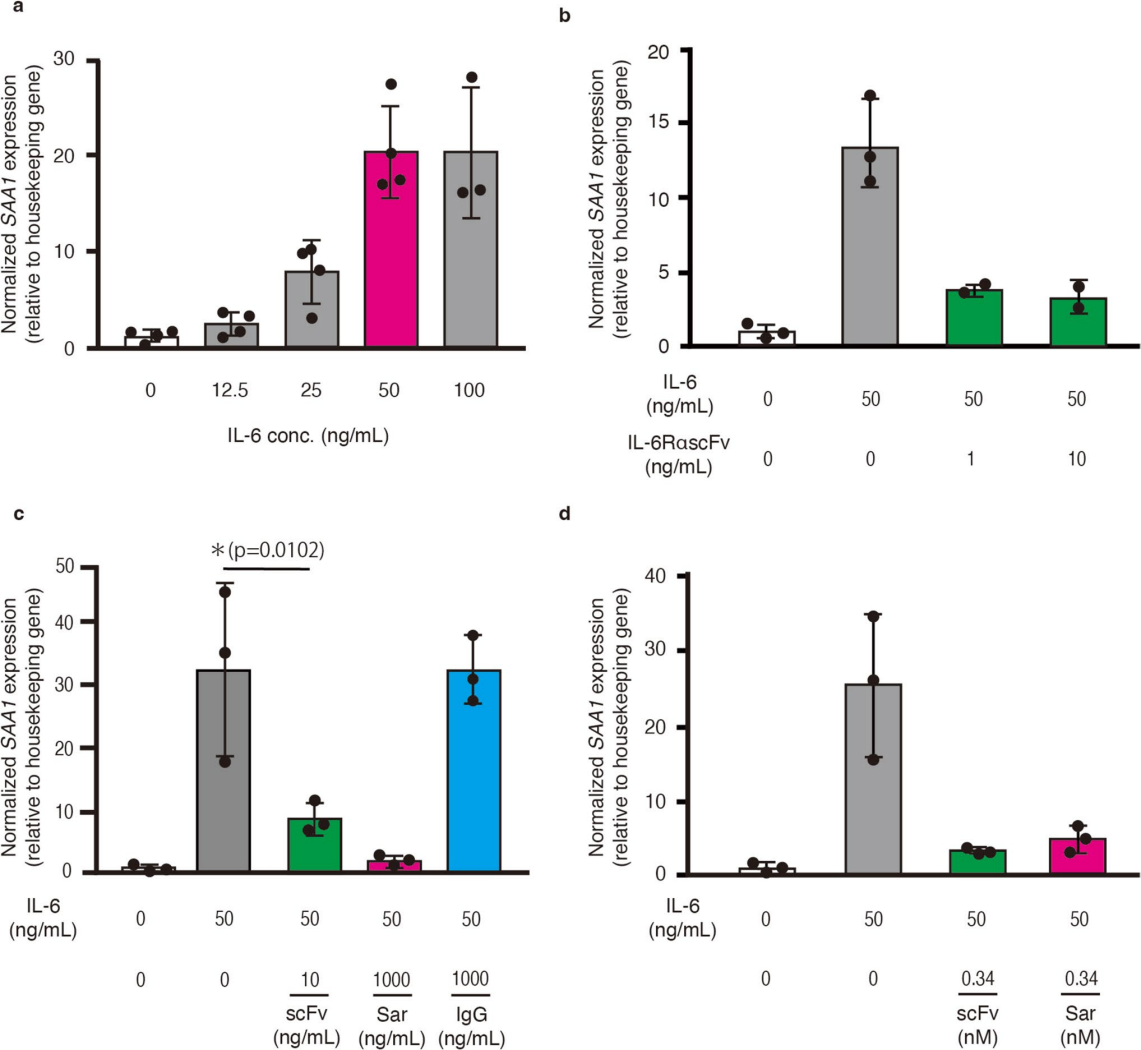
RT-qPCR results for the bioactivity of IL-6RαscFv in suppressing *SAA1* expression in HepG2 cells. **a** HepG2 cells were incubated with 0, 12.5, 25, 50, or 100 ng/mL of IL-6 for 24 h. **b** HepG2 cells were reacted for 30 min with 1 and 10 ng/mL of IL-6RαscFv purified from gmLAB, then stimulated with 50 ng/mL of hIL-6. **c** HepG2 cells were reacted for 30 min with IL-6RαscFv (10 ng/mL, green, scFv), sarilumab (positive control, 1000 ng/mL, red, Sar), and purified human IgG (negative control, 1000 ng/mL, blue, IgG). They were then stimulated with 50 ng/mL of hIL-6. **d** HepG2 cells were reacted with 0.34 nM of IL-6RαscFv (green, scFv) and sarilumab (red, Sar). Data are expressed as relative values versus the control (0 ng/mL of IL-6 and IL-6RαscFv). *:* p* < 0.05. Independent experiments were conducted more than three times, and representative data are shown

The ability of rIL-6RαscFv to suppress the inflammatory response was investigated using HepG2 human liver carcinoma cells. *SAA1* expression increased gradually in an IL-6 concentration-dependent manner, and the expression level was highest with 50 ng/mL of IL-6 (Fig. 4a). Thus, the subsequent bioactivity assay was performed using 50 ng/mL of IL-6. After 30 min of incubation with rIL-6RαscFv, cells were stimulated with IL-6 for 24 h. Initially, 1 and 10 ng/mL scFv were used for the validation to determine whether raising the scFv concentration could further suppress expression. It was found that 10 ng/mL scFv did not suppress more than 1 ng/mL scFv (Fig. 4b). The following bioactivity assay was conducted using 10 ng/mL scFv. The results showed that *SAA1* expression was significantly suppressed (*p* < 0.05) by 10 ng/mL of rIL-6RαscFv (Fig. 4c). Sarilumab (positive control, 1000 ng/mL) also suppressed *SAA1* expression. However, inhibition of *SAA1* expression was not observed with purified human IgG (negative control, 1000 ng/mL). In the comparative experiment (Fig. 4d), the *SAA1* expressions were inhibited to the same extent between rIL-6RαscFv and sarilumab at the same molar concentration (0.34 nM).

## Discussion

IL-6 is an important inflammatory cytokine involved in immune regulation, inflammatory responses, and chronic inflammation. It binds to a specific receptor, IL-6Rα, which exists as a transmembrane or soluble protein [36]. The IL-6/IL-6Rα complex then activates another transmembrane protein, Gp130, to act as a signal transducer of IL-6 [37]. This signaling activates the intracellular Janus kinase (JAK) mitogen-activated protein kinase (MAPK) [38] and JAK-signal transducer and activator of transcription 3 (STAT3) [39] signaling pathways. It affects transcriptional activity to finally modulate the expression of IL-6-responsive genes, such as acute-phase proteins [39]. Interestingly, a previous study demonstrated that IL-6 showed no binding affinity for gp130 in the absence of IL-6Rα [40], suggesting that IL-6/IL-6Rα binding is important for gp130 signal transduction. Thus, several mAbs that target IL-6 or IL-6Rα have been used to block these signaling pathways. Although some of these mAbs have shown beneficial therapeutic effects in various immunoinflammatory diseases, their high cost remains a significant limitation. Therefore, an attempt was made to construct new IL-6 signaling inhibitors that are more cost-effective.

The scFvs consist of the smallest functional units of an Ig that have antigen-binding activity, *i.e.,* the VH and VL chains, which are joined by a flexible peptide linker [17, 18]. These small antigen-binding scFvs offer several advantages, *e.g.,* they can be cleared from the blood much more rapidly than intact IgG [19], indicating that they have lower antigenicity. In addition, scFvs are simple and small peptides that can be easily expressed in functional form in genetically modified bacteria. In particular, gmLABs are an optimal platform for the production of scFvs for the treatment of diseases [25]. The use of gmLABs for the mucosal delivery of scFvs is cost-effective, because it does not require purification steps [22, 23, 26, 31]. In addition, there have been several gmLABs based on intracellular production reported so far, and their mucosal administration has shown good results [41], which are thought to be caused by the phagocytosis of immune cells and/or the rupture of gmLABs. We expect that intracellular IL-6RαscFv will attain biological activity by mucosal delivery of gmLABs. Thus, we established a gmLAB that synthesizes rIL-6RαscFv intracellularly in an attempt to develop a new biological strategy that could block IL-6/IL-6Rα signaling and thereby inhibit inflammatory diseases.

A NICE-based system for expressing rIL-6RαscFv was successfully constructed. The constructed NZ-IL6RαscFv cells were confirmed to produce rIL-6RαscFv under the control of nisin. As for optimization of IL-6RαscFv expression (Fig. 3b), IL-6RαscFv disappeared by 6 h. Interestingly, in previous studies of scFv-producing gmLABs [22, 23], recombinant scFvs were found for at least 24 h, indicating that scFvs produced by gmLABs are typically stable for at least 24 h. It was therefore thought that proteolysis may be the cause of the more rapid disappearance of IL-6RαscFv. The immunoreactivity assay of NZ-IL6RαscFv confirmed that rIL-6RαscFv binds to hIL-6Rα, but the ELISA signals were relatively low when compared with the IL-4scFv study [22]. Although these discrepancies might result from variations in protein solubility or codon use frequencies of expressed genes, the lack of research on this aspect is a limitation of this work.

In the immunoreactivity assay, there was some NZ-IL6RαscFv stuck on the plate, but the extent was minimal, indicating that this assay was valid. These data suggested its potential for suppressing inflammation in humans by inhibiting the interaction between hIL-6 and hIL-6Rα. In contrast, rIL-6RαscFv showed no detectable binding to mIL-6Rα, indicating minimal or no cross-reactivity with orthologous murine molecules (Supplementary material 1). A recent paper has reported that alanine mutation of Arg250, Arg252, Asp272, Gln296, and Phe298 of IL-6Rα substantially reduced or abolished the binding [42]. In the present study, comparison of the mouse and human amino acid sequences of IL-6Rα showed that these residues are mostly different (Supplementary material 4). This is thought to be the reason why IL-6RαscFv, which has a same complementary degerming region with sarilumab, does not cross-react with mouse IL-6Rα. This is a commonly encountered issue when evaluating human-specific therapeutic Abs in non-primate models, since the Abs often lack efficacy in experimental animals, such as mice and rats [43].

To further investigate the inhibitory properties of rIL-6RαscFv on IL-6/IL-6Rα signaling, a competitive ELISA to assess the ability of rIL-6RαscFv to block IL-6 binding to hIL-6Rα was performed. First, the measurements were performed using positive and negative controls, and it was confirmed that the inhibition assay was established (Fig. 3d). The rIL-6RαscFv showed a concentration-dependent decrease in absorbance, indicating successful inhibition of the binding. However, since its capacity to suppress inflammatory responses remained unclear, an inflammatory inhibition assay using human HepG2 liver carcinoma cells was also performed [44]. In humans, after IL-6 is synthesized in a local lesion at the initial stage of inflammation, it rapidly induces hepatocytes to produce acute-phase proteins, such as C-reactive protein, SAA, fibrinogen, haptoglobin, and α1-antichymotrypsin. SAA is recognized as a biological marker of an inflammatory response to viral infections, including severe acute respiratory syndrome coronavirus 2 infection [45, 46], vaccination [47], or traumatic injury [48]. Hepatic SAA1 is regulated by inflammatory cytokines, such as IL-6, IL-1β, and tumor necrosis factor-α [49, 50]. In the present study, the *SAA1* expression level was used as an indicator to evaluate the effectiveness of rIL-6RαscFv in inhibiting inflammation in HepG2 cells. The relative *SAA1* expression level was significantly suppressed after 30 min of incubation with rIL-6RαscFv, indicating that rIL-6RαscFv inhibited the inflammatory response. Finally, a comparative experiment with sarilumab was performed to evaluate the efficacy of the scFv. As a result, rIL-6RascFv was shown to suppress *SAA1* expression to the same extent as sarilumab at 0.34 nM. Interestingly, a recent report suggested that whether therapeutic Abs bind near the IL-6-binding site is very important for the inhibition of IL-6/IL-6R signaling [42]. Based on the results of this comparative experiment, it was inferred that scFv and sarilumab bind to a similar part of IL-6Rα. In addition, the present 3D structure prediction study provided insights into the inhibition efficacy of rIL-6RαscFv (Supplementary material 5). In complex 1, rIL-6RαscFv binds to the region D1 to D2 in IL-6Rα, whereas in complexes 2 and 3, rIL-6RαscFv binds to the region D2 to D3 in IL-6Rα. In complex 2, rIL-6RαscFv binds to residue S228 of IL-6Rα located at L5 of the IL-6-binding site, and in complex 3, rIL-6RαscFv binds to L108 (L1), D101 (L5), E278 (L7), H256 (L6), and S228 (L5) of IL-6Rα, suggesting that both complexes would inhibit IL-6/IL-6R signaling more efficiently than complex 1. Since it had the same inhibitory ability as sarilumab, which interacts with the D2 and D3 domains of IL-6Rα [42], scFv is thought to bind to the D2 or D3 regions of hIL-6Rα, such as complex 2 and 3.

Although the inflammation inhibitory effects of NZ-IL6RαscFv were demonstrated in the present in vitro experiments using purified rIL-6RαscFv, the effects were not examined in mouse models using engineered bacteria, because no cross-reactivity was seen with mIL-6Rα. Therefore, further investigations of the effects of NZ-IL6RαscFv are warranted in a genetically humanized mouse model, in which the endogenous *IL-6R* gene is replaced by the *hIL-6R* gene [51] to develop bacterial delivery of IL-6RαscFv. Specifically, the efficacy of this bacterial delivery should be evaluated in chronic inflammatory diseases such as rheumatoid arthritis [52], which are correlated with failure of IL-6 control. In addition to the evaluation of pathology, cytokine levels, and swollen paws, the measurement of blood SAA1 concentrations will also be needed.

## Conclusions

A genetically modified strain of *L. lactis* NZ9000, NZ-IL6RαscFv, which produces rIL-6RαscFv under optimized culture conditions, was successfully developed. The present findings demonstrated the potential of NZ-IL6RαscFv for treating inflammatory diseases caused by IL-6/IL-6Rα signaling.

## Supplementary Information

Below is the link to the electronic supplementary material.

Supplementary file1 (DOCX 3905 KB)
