# Supplementary material for: Development of a Genetically Modified Lactococcus lactis Strain that Produces a Single-Chain Variable Fragment Targeting Interleukin-6 Receptor α to Suppress Serum Amyloid A
Source: Curr Microbiol. 2025 Sep 26;82(11):522. doi: 10.1007/s00284-025-04518-1 (PMC12474681; doi:10.1007/s00284-025-04518-1)
Supplement: Supplementary file 1 — Supplementary file1 (DOCX 3905 KB) [file 284_2025_4518_MOESM1_ESM.docx]

**Supplementary materials**

| **Sample** | **Concentration** | **Absorbance** | **Mean** |
| --- | --- | --- | --- |
| PBS | - | 0.052 | 0.052 |
|  |  | 0.052 |  |
| NZ-VC | 2 mg/mL  (total protein conc.) | 0.058 | 0.057 |
|  |  | 0.055 |  |
| NZ-IL6RαscFv | 2 mg/mL  (total protein conc.) | 0.054 | 0.053 |
|  |  | 0.051 |  |
| Mouse IL-6  (His conjugate) | 5 μg/mL | 0.112  0.115  0.109  0.107 | 0.111 |

**Supplementary material 1. Immunoreactivity of NZ-IL6RαscFv to mIL-6Rα**

Immunoreactivity assays of NZ-IL6RαscFv, NZ-VC cellular extracts, and His-tag conjugated mouse IL-6Rα to mIL-6Rα were performed. **a** A schematic representation of the ELISA-based technique. **b** Absorbance of the immunoreactivity assay. Solid phase-immobilized mIL-6Rα was reacted with 2 mg/mL of gmLAB cell extract (NZ-IL6RαscFv or NZ-VC). Then, 5 μg/mL of His-tag conjugated mouse IL-6 (Acro Biosystems, Newark, DE, USA) were used as a positive control. The absorbance remained at the baseline level for the cellular extracts of NZ-IL6RαscFv and NZ-VC. These data suggest that rIL-6RαscFv has minimal or no cross-reactivity with orthologous murine molecules.


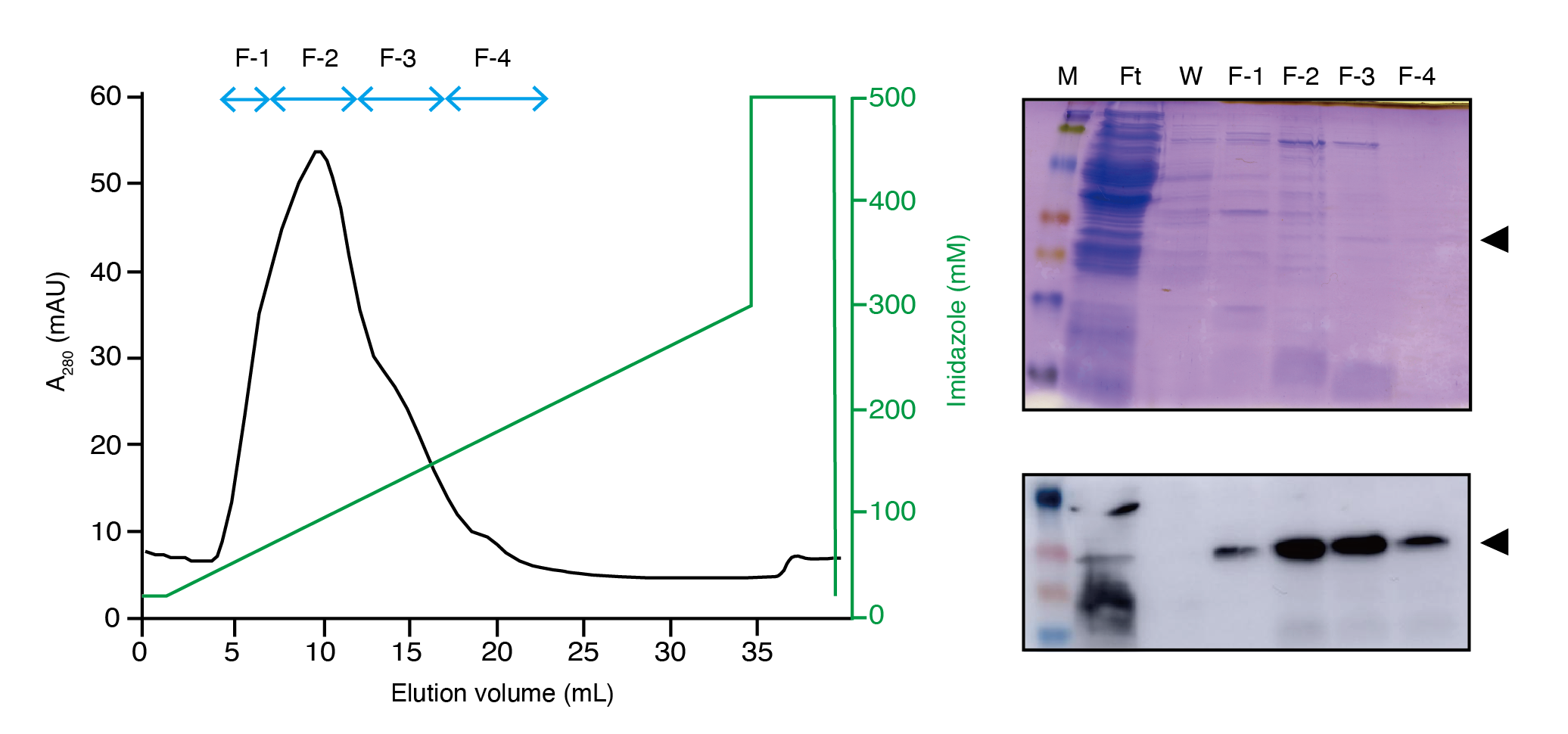


**Supplementary material 2. Purification of rIL-6RαscFv**

The rIL-6RαscFv was purified from cellular extracts prepared from nisin-induced NZ-IL6RαscFv by an immobilized metal ion affinity chromatography technique. **a** A chromatogram in the elution phase of chromatography. The eluent was collected in four fractions (F-1 to F-4). **b** Fractions obtained from chromatography were analyzed with SDS-PAGE, CBB-staining (upper), and western blotting using an anti-His-tag Ab (lower). The arrows show the rIL-6RαscFv (29.1 kDa). The purest fraction, F-4, was used for further investigation.

**a**

| **Concentration**  **(ng/mL)** | **Absorbance** | **Mean** | **SD** |
| --- | --- | --- | --- |
| 0 | 1.417  1.399 | 1.408 | 0.013 |
| 1 | 1.335  1.381 | 1.358 | 0.033 |
| 3 | 1.218  1.273 | 1.245 | 0.039 |
| 9 | 0.756  0.768 | 0.762 | 0.008 |
| 27 | 0.362  0.359 | 0.360 | 0.002 |
| 81 | 0.187  0.192 | 0.190 | 0.004 |
| 243 | 0.097  0.089 | 0.093 | 0.006 |
| 729 | 0.062  0.067 | 0.064 | 0.004 |

**b**

| **Samples** | **Absorbance** | **Mean** | **Concentration**  **(ng/mL)** | **SD** |
| --- | --- | --- | --- | --- |
| Mouse IL-6  (His conjugate)  50 ng/mL | 0.273  0.277 | 0.275 | 36.070 | 0.424 |
| purified human IgG  500 ng/mL | 1.355  1.337 | 1.346 | 1.116 | 0.177 |
| rIL-6RαscFv  F-4, 10-fold dilution | 0.385  0.393 | 0.389 | 24.238 | 0.411 |

**Supplementary material 3. Determination of the concentration of purified rIL-6RαscFv**

After purification of rIL-6RαscFv, its concentration was measured using competitive ELISA (His Tag ELISA Detection Kit, Funakoshi). Purified human IgG (Fujifilm Wako Pure Chemicals, 500 ng/mL) and His-tag conjugated mouse IL-6 (Acro Biosystems, 50 ng/mL) were used as a negative control and positive control, respectively, to validate the competitive assay. The experiment was conducted according to the manufacturer's instructions. **a** 0, 1, 3, 9, 27, 81, 243, 729 ng/mL of His-tagged protein that is included in the reagent provided by the manufacturer was used as the standard of the competitive ELISA. **b** The results of the positive and negative control samples (36.070 and 1.116 ng/mL) suggested that competitive ELISA was established (The detection limit: 1 ng/mL). The concentration of 10-fold diluted rIL-6RαscFv, F-4, was measured. Multiplying the measured concentration (24.238 ng/mL) by 10, the concentration of rIL-6RαscFv was determined to be 242 ng/mL. The standard curve and concentrations of the sample were determined using Microplate Manager 6 (Bio-Rad)

**a**

| **Sarilumab** | **IL-6Rα** | |
| --- | --- | --- |
|  | **H. sapiens** | **M. musculus** |
| Trp53 (H) | Arg250 | Leu |
| Asn54 (H) | Phe248 | Tyr |
| Arg57 (H) | Glu296 | Glu |
| Arg100 (H) | Asp272 | Tyr |
| Trp32 (L) | Arg252 | Gln |
| Phe94 (L) | Phe298 | Leu |

**b**

**Supplementary material 4. Comparison of the binding sites of human and mouse IL-6Rα to sarilumab**

To consider the differences in immunoreactivities of human and mouse IL-6Rα to sarilumab, the amino acid sequences were compared using Clustal Omega. **a** The amino acid sequences of human and mouse IL-6Rα from 240 to 299 residues are depicted. The amber color indicates the important residues for sarilumab binding (ref). Red-highlighted amino acids in mouse IL-6Rα show the residues different from human IL-6Rα. **b** The residues that are correlated to sarilumab/IL-6Rα interaction. Left and middle columns: The residues of sarilumab that are involved in the binding to human IL-6Rα. H and L indicate heavy chain and right chain, respectively. Right column: The mouse IL-6Rα residues corresponding to those of human IL-6Rα are shown. Only glutamic acid, which is involved in arginine 57 of the sarilumab heavy chain, has the same residue in mouse IL-6Ra as it does in human IL-6Rα (underlined).

**Supplementary material 5. *In silico* characteristics of the binding of rIL-6RαscFv to IL-6Rα**

To better understand how rIL-6RαscFv (Fig. 1c) binds to IL-6Rα, the structures of rIL-6RαscFv/IL-6Rα complexes were predicted using a protein-protein docking algorithm.

**a** Cartoon representation of the IL-6Rα extracellular structure (Protein Data Bank identifier: 1N26). IL-6Rα (green) consists of D1 (residues 1-93), D2 (residues 94-194), and D3 (residues 195-299) domains. The loops L1–L7 (cyan; L1–L7 consist of the polypeptides S106–N110 (L1), K133–P138 (L2), A160–F168 (L3), Q190–G193 (L4), S227–R233 (L5), M250–H256 (L6), and Q276–Q281 (L7)) that can interact with IL-6 around the juncture of D2 and D3 are highlighted. **b** The interface between IL-6RαscFv and IL-6Rα. In total, 500 structures were obtained; the top three candidate structures are depicted as complexes 1 to 3. The VH, VL, and peptide linker region of IL-6RαscFv are shown in blue, yellow, and red, respectively. The IL-6Rα- and IL-6-binding sites are shown in green and cyan, respectively. The yellow dashed lines indicate hydrogen bonds.

Complex 1 indicated that, in IL-6RαscFv, residues R100 (VL) and Y180 (VH) bind to residues P46, A47, and A48, which are in the Domain (D) 1 region of IL-6Rα. It also indicated that, in the VH of IL-6RαscFv, residue R100 binds to A47 of IL-6Rα. In addition, in the VL of IL-6RαscFv, residues R149, Y180, G197, and S198 bind to residues E151, P46 and A48, Y148, and T125, respectively, which are in the D2 region of IL-6Rα, but not in the IL-6-binding site. Thus, all of the binding sites are in the D1 or D2 region, but not in the IL-6-binding site.

Complex 2 indicated that, in the VH of IL-6RαscFv, residues Y60 and K65 bind to residue S228 of IL-6Rα, which is located in loop (L) 5 of the IL-6-binding site (Fig. 5a, b).

Complex 3 indicated that, in the VH of IL-6RαscFv, residues R57 and D101 bind to residues L108 (L1) and S228 (L5) of IL-6Rα; moreover, in the VL of IL-6RαscFv, residues S162, Y180, and Y227 bind to residues E278 (L7), H256 (L6), and S228 (L5), respectively, of IL-6Rα.
